# Supplementary material for: DMRforPairs: identifying Differentially Methylated Regions between unique samples using array based methylation profiles
Source: BMC Bioinformatics. 2014 May 15;15:141. doi: 10.1186/1471-2105-15-141 (PMC4046028; doi:10.1186/1471-2105-15-141)
Supplement: Additional file 2 — DMRforPairs output for the comparison of A431-MCF7 and NA17018-NA17105. Please start from the HTML files in each folder. Available via the BMC Bioinformatics website. [file 1471-2105-15-141-S2.zip › 1394847754114233_MOESM2_ESM/A431_MCF7/figures/11471.pdf]

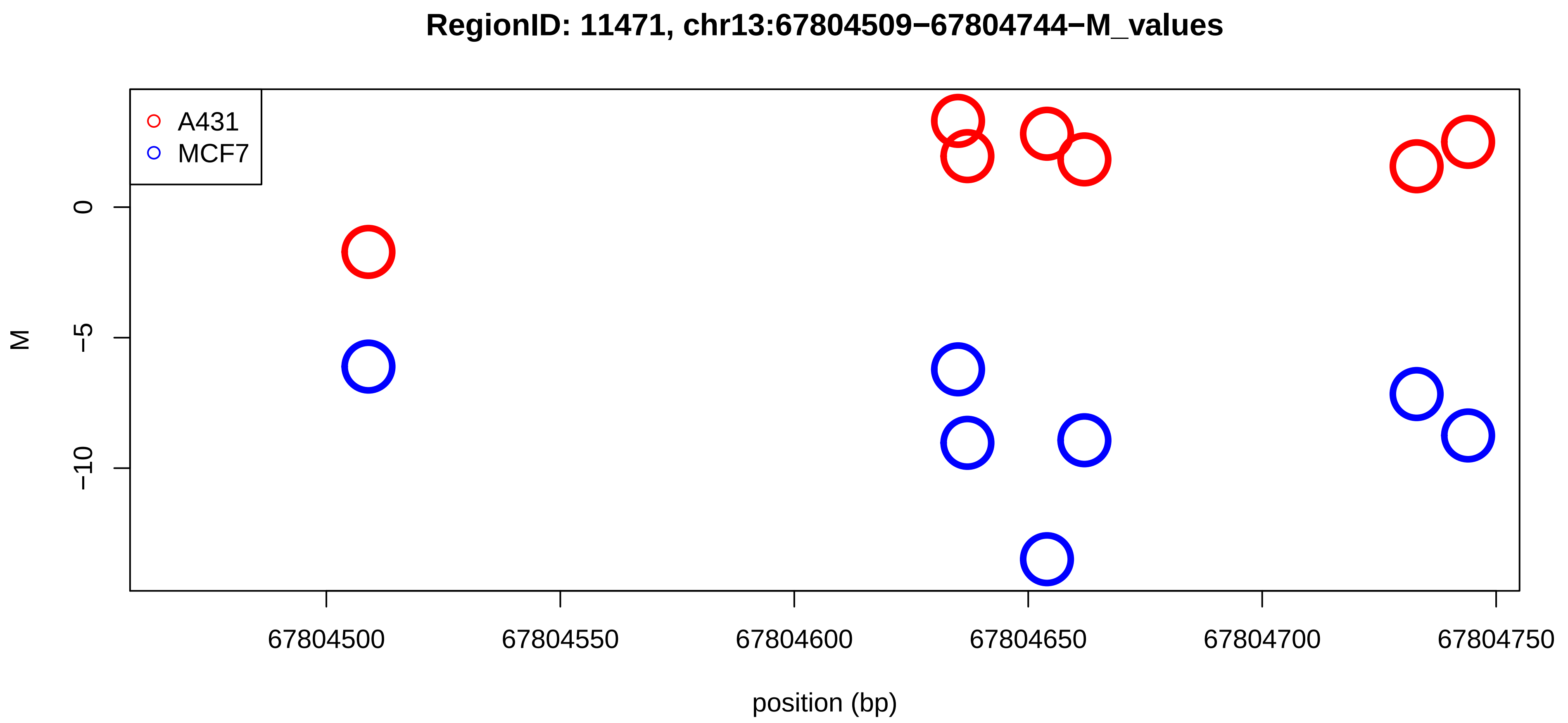

RegionID: 11471, chr13:67804509−67804744−M_values
A431
MCF7
M
0
−5
−10
67804500
67804550
67804600
67804650
67804700
67804750
position (bp)

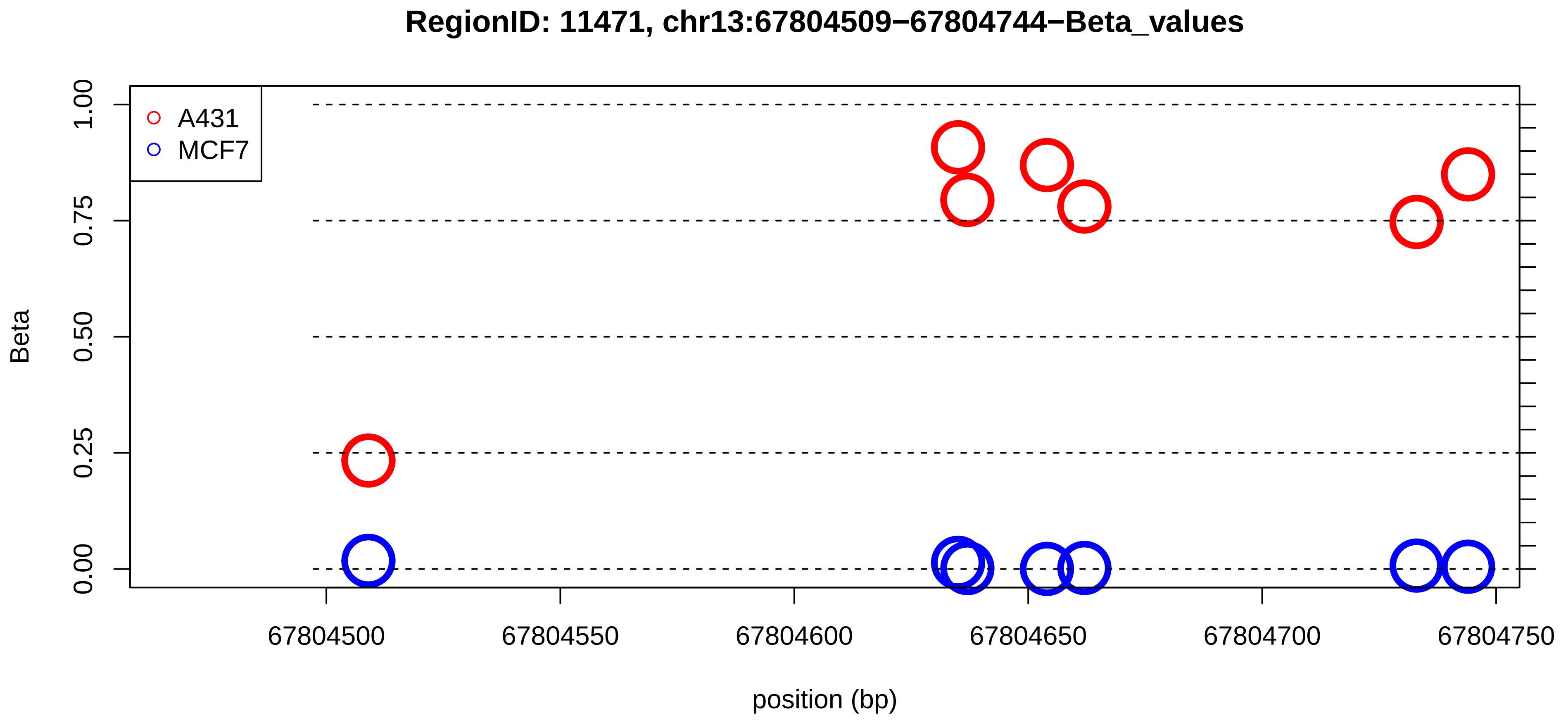

RegionID: 11471, chr13:67804509−67804744−Beta_values
A431
MCF7
Beta
0.00
0.25
0.50
0.75
1.00
67804500
67804550
67804600
67804650
67804700
67804750
position (bp)

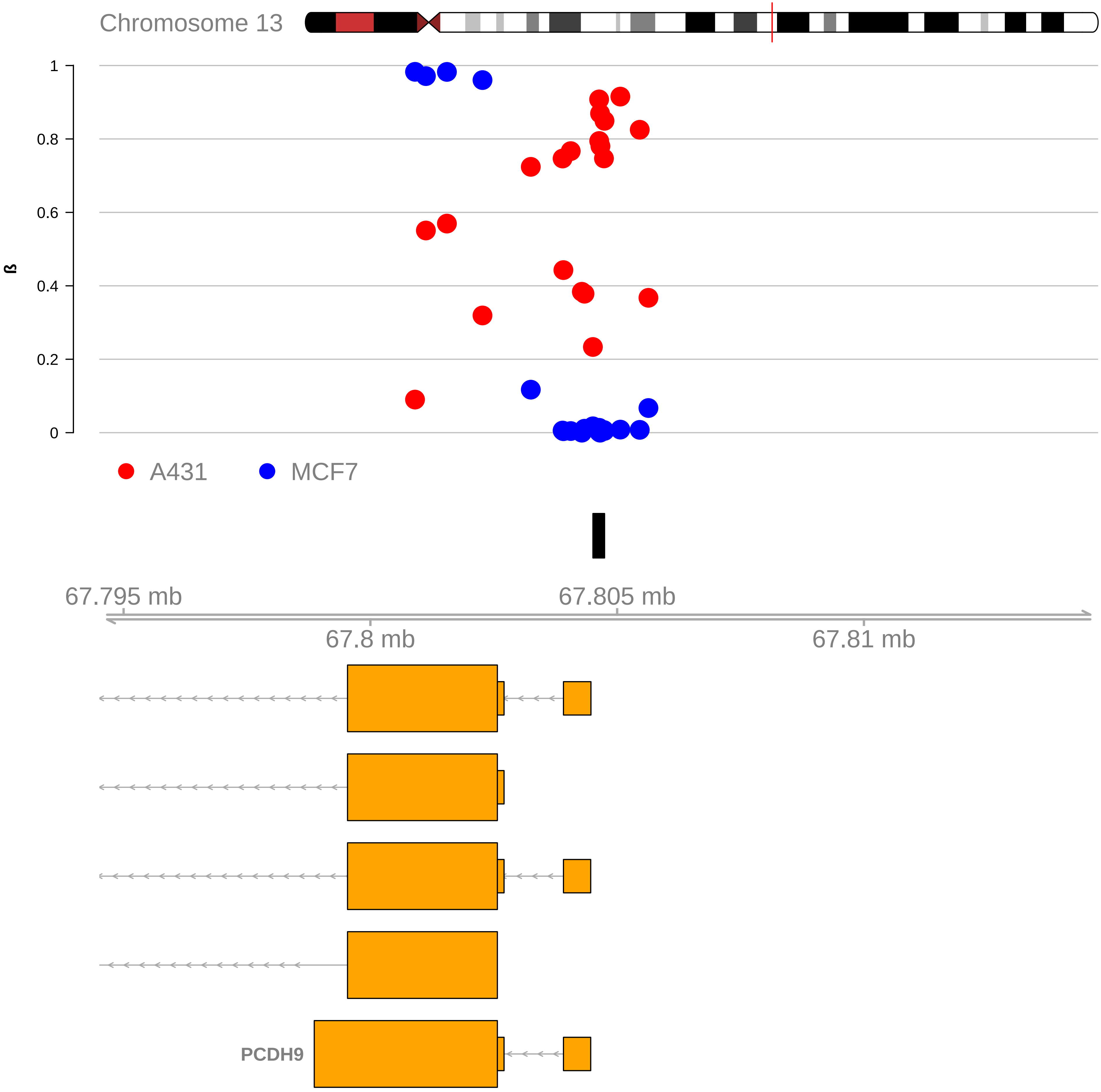

Chromosome 13
ß
1
0.8
0.6
0.4
0.2
0
A431
MCF7
67.795 mb
67.805 mb
67.8 mb
67.81 mb
PCDH9
